# Early selection of carrot somatic hybrids: a promising tool for species with high regenerative ability

**DOI:** 10.1186/s13007-023-01080-4

**Published:** 2023-10-07

**Authors:** Katarzyna Mackowska, Katarzyna Stelmach-Wityk, Ewa Grzebelus

**Affiliations:** Department of Plant Biology and Biotechnology, Faculty of Biotechnology and Horticulture, University of Agriculture in Krakow, Mickiewicza 21, 31-120 Krakow, Poland

**Keywords:** *Daucus carota L.*, Dual-labelling, Electrofusion, ILP markers, Micromanipulation, Protoplasts

## Abstract

**Background:**

Since its discovery, somatic hybridization has been used to overcome the sexual barriers between cultivated and wild species. A combination of two somatic cells might provide a novel set of features, often of agronomical importance. Here, we report a successful approach for production and selection of interspecific somatic hybrid plants between cultivated and wild carrot using dual-labelling of protoplasts and an early selection of fused cells via micromanipulator. Both subspecies used in this study are characterised by a very high regenerative ability in protoplast cultures. Thus, a precise and effective method of hybrid selection is essential to assure the development and regeneration of much less numerous heterokaryons in the post-fusion cell mixture.

**Results:**

Electrofusion parameters, such as alternating current and direct current, were optimised for an efficient alignment of protoplasts and reversible membrane breakdown followed by a cell fusion. Four hundred twenty-nine cells emitting green–red fluorescence, identified as hybrids, were obtained. Co-culture with donor-derived protoplasts in the alginate feeder layer system stimulated re-synthesis of the cell wall and promoted cell divisions of fusants. Somatic embryogenesis occurred in hybrid-derived microcalli cultures, followed by plant regeneration. Regenerated hybrids produced yellowish storage roots and leaves of an intermediate shape between cultivated and wild subspecies. The intron length polymorphism analysis revealed that 123 of 124 regenerated plants were hybrids.

**Conclusions:**

The developed protocol for protoplast fusion and an early selection of hybrids may serve as an alternative to combining genomes and transferring nuclear or cytoplasmatic traits from wild *Daucus* species to cultivated carrot.

## Background

Somatic hybridization is a technique that may combine somatic cells of two different varieties, species, or genera, to generate new variability. In this way, as a result of bypassing pre- and post-zygotic barriers, plants (called somatic hybrids) with a unique set of features can be developed [1]. For that reason, somatic hybridization is of particular interest to plant breeders in the context of introducing/generating agronomically important traits such as cytoplasmic male sterility (CMS) or resistance to biotic or abiotic stresses. Thus, the long process of homozygous line backcrossing during the production of hybrid cultivars can be shortened. It is particularly valuable for biennial crops belonging to the Apiaceae family, such as carrot, celery, parsley or parsnip. Over the years, the successful development of somatic hybrids for both ornamental and crop species, including those belonging to the *Daucus* genus, has been reported [2–8]. Despite observed progress in the production of somatic hybrids, somatic hybridization is routinely used for the improvement of existing genetic resources of only a few crops [5, 9, 10]. This is most likely due to the fact that the development of somatic hybrids is a multi-stage process and often requires detailed optimization of subsequent stages.

So far, successful protocols for protoplast isolation, fusion and plant regeneration from protoplast cultures of economically important members of the Apiaceae family such as carrot and celery, have been described, both for different source tissues [11–15] and for various wild and cultivated species and subspecies [8, 14, 16–22].

Since the first report of a successful isolation of plant protoplasts [23] and protoplast-to-plant regeneration [24], an increased interest in developing an efficient method for protoplast fusion was observed. Among numerous chemical compounds tested, the use of polyethylene glycol (PEG) proved to be effective for animal, bacterial and plant cell fusions [25–28]. Zimmermann and Scheurich [29] performed the electrically stimulated fusion of *Vicia faba* protoplasts, whereas, four years later Bates and Hasenkampf [30] produced somatic hybrid plants from electrofused protoplasts of *Nicotiana tabacum* and *N. plumbagnifolia*. This method of fusion, when carefully adjusted to the species of interest, proves to have less of an immediate effect on cell viability than PEG-mediated techniques. Moreover, once optimized for the species of interest, electrofusion is generally easy to perform than most alternative methods. This method exploits the alignment, and therefore, a close contact of protoplasts that is obtained by the application of alternating current (AC). Then, by applying short pulses of direct current (DC), a reversible disintegration of the cell membrane is forced, leading to the fusion of protoplasts [29]. The parameters of applied AC and DC, such as voltage and time of exposure are critical factors to be considered in the process of optimization of electrofusion. Regardless of the method used for protoplast fusion, it is advisable to implement an early selection step including the selection of heterokaryons from the post-fusion mixture to avoid unnecessary and labour-intensive regeneration of non-fused cells [31]. However, this step seems to be more feasible after electrofusion than after PEG-mediated fusion, where due to the properties of PEG, the cells often aggregate in the post-fusion mixture and adhere firmly to the bottom of the Petri dish. Such early selection step is particularly true in case of species characterized by a high regenerative capacity in protoplast cultures, such as wild and cultivated carrots. The use of various non-toxic and stable fluorescent dyes, such as tetramethylrhodamine isothiocyanate, fluorescein isothiocyanate or fluoresceine diacetate [32], to differentially label protoplasts derived from both parental components, as shown for *Phaseolus*, *Pisum* and *Lathyrus* [33, 34], may significantly aid early selection of obtained fusants from the post-fusion cell mixture [31]. Vital staining allows for fast protoplast discrimination and identification of hybrid cells characterised by dual-colour fluorescence.

Regardless of an early selection of putative hybrid cells, the use of somatic hybridization in crop improvement requires an effective and efficient system of markers for the validation of a hybrid status of regenerated plants, tailored to the species of interest. The use of molecular markers for the detection of DNA polymorphism in somatic hybrids has its advantages as it allows for identification of hybrids at the culture stage, even after incomplete regeneration, leading to the reduction of resources, space and time required for production of somatic hybrids. Depending on the type of protoplast fusion, i.e. symmetric or asymmetric, one can develop markers suited for identification of polymorphisms in either organelle genomes and/or the nuclear genome. The use of DNA polymorphism resulting from the activity of transposable elements (TEs) for the characterization of germplasm has been documented for many species, e.g. banana, bamboo, barley, carrot, rice or wheat [35–40]. Codominant markers exploiting TEs insertional polymorphism might serve as a tool for validation of hybrids resulting from the symmetric protoplast fusion event. It allows for identification of heterozygotes originating from the fusion of protoplasts derived from both parental lines, providing that donors were opposite homozygotes at a given TE insertion site. Once developed and validated, the marker system is usually readily available, relatively time- and cost-efficient, and can be used at an early growth stage of a putative hybrid.

In this paper, we report a successful method for the production and selection of interspecific somatic hybrids between *D. carota* subsp. *sativus* and *D. carota* subsp. *gadecaei* using differential fluorescent labelling of protoplasts for the manual selection of putative hybrid cells via micromanipulator from the post-fusion mixture directly after the completion of protoplast fusion. The objectives of the study were as follows: (1) selection of fluorescent labels for the effective detection of heterofusion products; (2) optimization of the electrofusion procedure; (3) development of an efficient system for the regeneration of hybrids; (4) development of a molecular marker-based system for the validation of the hybrid nature of regenerated plants.

## Results

### Development of dual fluorescence labelling of protoplasts for the effective detection of heterofusion products

**Fig. 1:**
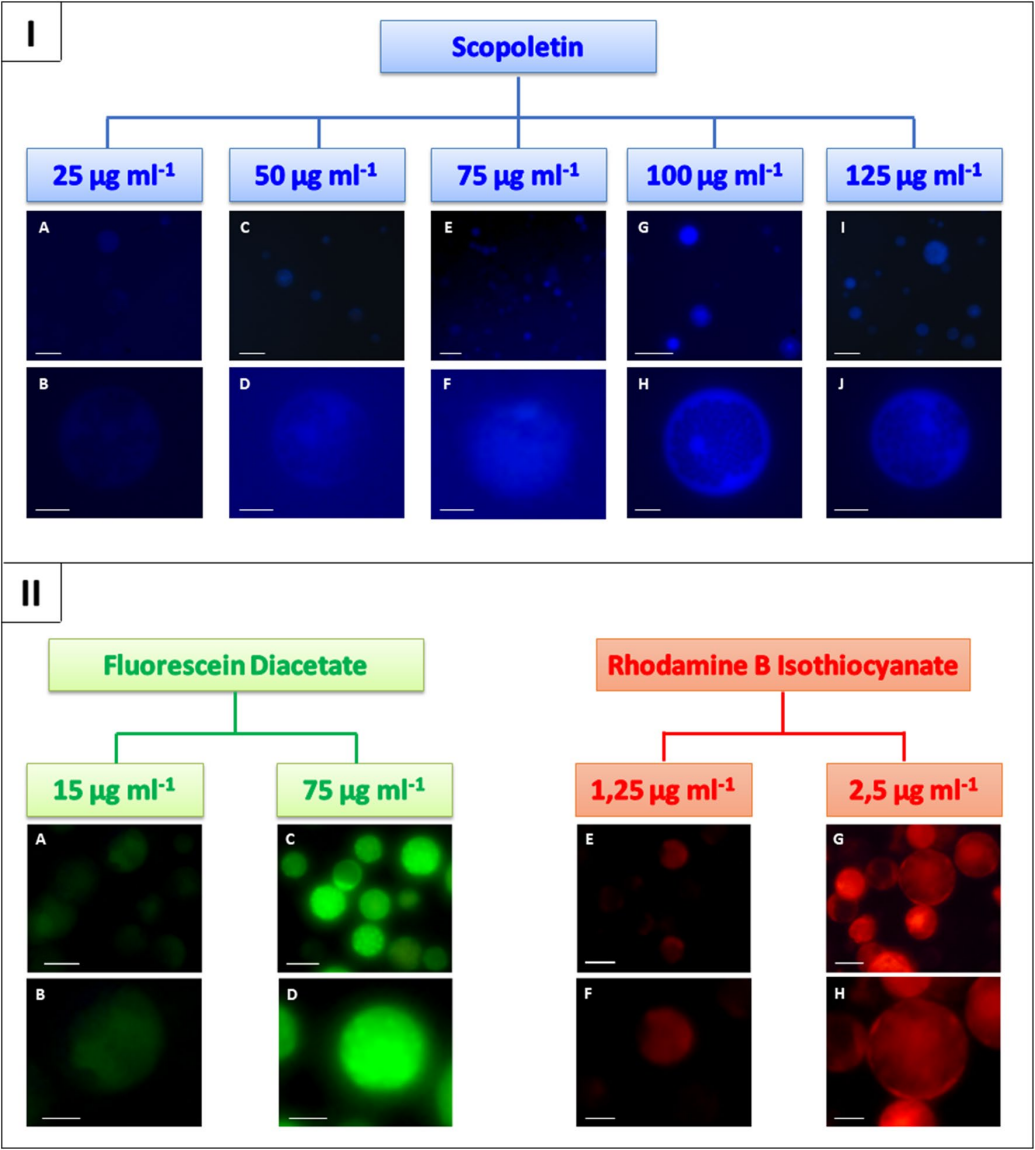
Intensity of protoplast fluorescence after staining with different concentrations of: (**I**) scopoletin, blue fluorescence (**A**-**J**), scale bars: 10 µm (**B**, **D**, **F**, **H**, **J**), 50 µm (**A**, **C**, **E**, **G**, **I**); (**II**) fluorescein diacetate, green fluorescence (**A**-**D**) and rhodamine B isothiocyanate, red fluorescence (**E**–**H**), scale bars: 10 µm (**B**, **D**, **F**, **H**), 20 µm (**A**, **C**, **E**, **G**)

Three fluorochromes with different spectral properties were used to label protoplasts i.e. fluorescein diacetate (FDA), rhodamine B isothiocyanate (RBITC) and scopoletin. The intensity of the blue fluorescence emitted by protoplasts after overnight incubation with scopoletin in the enzyme mixture was dependent on the applied dye concentration (Fig. 1I). The use of concentrations in the range from 25 to 75 µg ml^−1^ did not provide a homogenous staining of all protoplasts and significant differences in the intensity of blue fluorescence were noted. For some of the cells, blue fluorescence was not observed, while others showed a weak signal. Additionally, after a prolonged exposure to UV light, the quenching of fluorescence and the red autofluorescence of chlorophyll were also observed. The use of scopoletin in the concentrations of 100 µg ml^−1^ and 125 µg ml^−1^ allowed for an appropriate labelling of protoplasts. Slightly less intense fluorescence was observed in the areas of the cells in which chloroplasts were located. Similarly, in case of FDA- and RBITC-stained protoplasts the intensity of green and red fluorescence, respectively, was dependent on the concentration of applied dye (Fig. 1II). Higher concentrations of FDA (75 µg ml^−1^) and RBITC (2.5 µg ml^−1^) were shown to induce optimal levels of fluorescence in stained protoplasts, while lower concentrations resulted in very poor fluorescence, thereby preventing cell identification. The intensity of fluorescence after staining protoplasts of *D. carota* subsp. *gadecaei* with higher concentrations of FDA and RBITC was similar to the intensity observed in ‘Dolanka’ protoplasts. In the next step, a proper purification system of the stained protoplast suspensions from unbound dye particles was developed. Mixing of FDA- and RBITC-stained protoplast suspensions after two washes in cold mannitol resulted in green–red fluorescence of some protoplasts. It proved that unbound dye particles present in the protoplast suspension penetrated into the already stained protoplasts and, as a result, dual fluorescence of the cells was observed. Such cross-stained protoplasts were not observed if protoplast suspensions were washed three times in cold mannitol solution. However, after four washes fluorescence emitted by protoplasts was very low. In the case of scopoletin, most of unbound dye particles were removed during protoplast isolation and purification procedure. Still, double washing of scopoletin-stained protoplast suspension in cold mannitol was necessary to remove K^+^, Na^+^, Ca^2+^ ions coming from solutions used during the protoplast isolation procedure. The presence of such ions in protoplast suspensions is disadvantageous, resulting in reduction of fusion buffer conductivity and thus the efficiency of fusion. However, such additional washes resulted in the dye being washed out of the cells and consequently the intensity of blue fluorescence had been reduced. Thus, identification of scopoletin-stained cells was limited. Therefore, for further study, FDA and RBITC have been used in the procedure of differential labelling of parent protoplasts before fusion experiments. Scopoletin has been eliminated from further study due to low dye stability in cells and fast quenching fluorescence compared to FDA and RBITC.

**Fig. 2:**
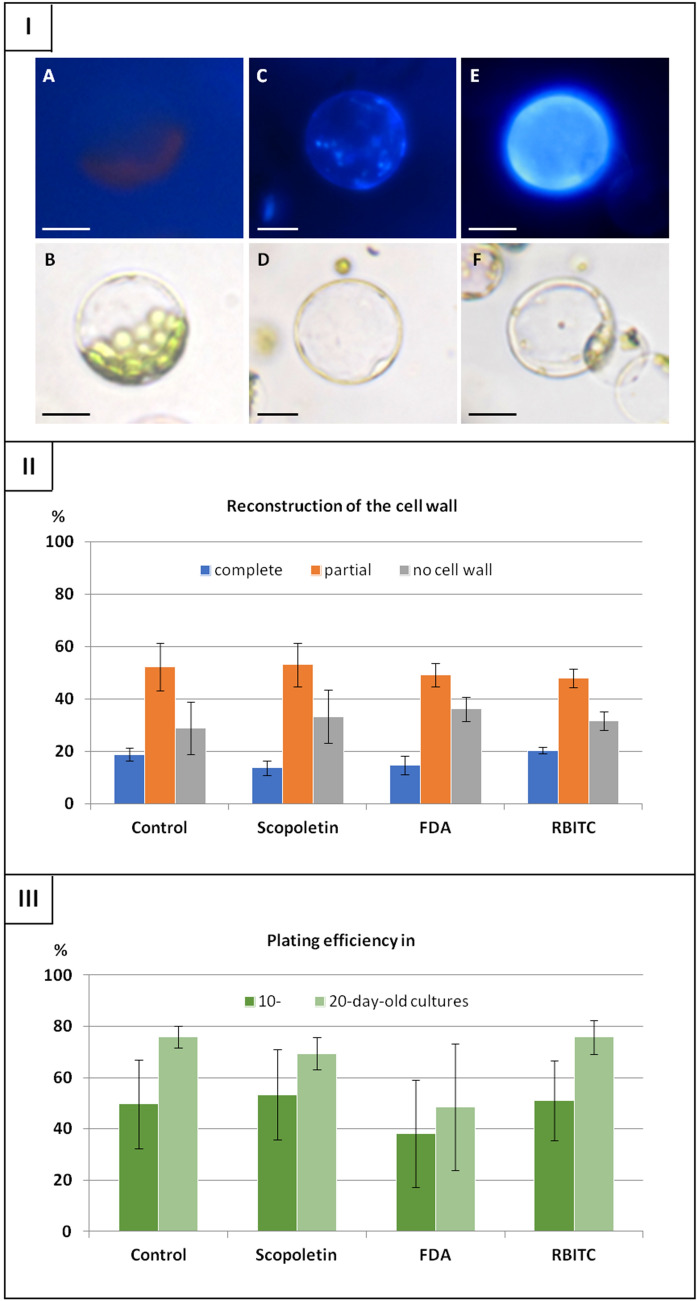
Effect of fluorochromes on development of ‘Dolanka’ protoplast cultures. (**I**) **A**, **B**) fresh isolated protoplast with completely removed cell wall, (**C**, **D**) cell with partially and (**E**, **F**) completely reconstituted cell wall; (**A**, **C**, **E**) calcofluor-stained cells and (**B**, **D**, **F**) their image under bright-field illumination. (**II**) Frequency of cells with completely reconstructed cell walls examined after calcofluor staining of cellulose in 48 h-old cultures. (**III**) Plating efficiency in 10- (**A**) and 20-day-old (**B**) protoplast cultures. *FDA* fluorescein diacetate, *RBITC *rhodamine B isothiocyanate. Bars represent means ± SE obtained from three independent experiments. Statistically significant differences between variants were not observed both within particular categories of cell wall reconstitution (**II**) and within subsequent time points of plating efficiency (**III**). Scale bar: 20 µm

Both, control and fluorochrome-treated protoplasts showed ability to completely re-synthesize the cell wall. The evidence for the presence of the cell wall was blue fluorescence of cellulose visible after calcofluor treatment (Fig. 2I). In 48 h-old cultures, cells with a complete and partially re-constituted wall, as well as cells without the cell wall were observed. Approximately 17% of cells showed intense blue fluorescence, however no significant effect of applied fluorochromes on cell wall reconstruction in comparison to control protoplasts was observed (P = 0.3). Number of cells with completely reconstituted cell walls varied from 14% for scopoletin- to 20% for RBITC-stained protoplasts (Fig. 2II). In the following days, typical changes in the shape and reorganization of cell organelles were observed both in control and fluorochrome-treated cultures. In consequence, cells re-entered mitotic divisions and multicellular aggregates were formed. In 10-day-old cultures no differences in number of cell aggregates (expressed by plating efficiency) were observed, regardless of the culture variant (P = 0.9). At this time point, the plating efficiency in the control cultures reached 49%, while in fluorochrome-treated cultures varied from 38 to 53% (Fig. 2III). Over time, the number of cell aggregates increased, in 20-day-old cultures on average by 20% in comparison to 10-day-old cultures. Similarly to 10-day-old cultures, the negative effect of applied fluorochromes on the formation of cell aggregates was not observed (P = 0.5). At both time points, FDA-treated cultures showed slightly lower planting efficiency compared to the control, but the differences were statistically not significant. Both, control and fluorochrome-treated cultures showed ability to regenerate into plants. Regardless of the culture variant, no differences in the number of regenerated plants were noticed.

### Establishment of thin alginate feeder layer system for development of manually selected protoplasts

During the transfer of manually selected protoplasts from the transfer needle of the micromanipulator to the alginate droplets, the agglutination, disintegration, and loss of cells were observed. Only 50% of cells embedded in the alginate matrix showed proper morphology and undamaged membranes. The developmental responses were observed only for the cells co-cultured with a thin alginate feeder layer (Table 1). It was found that progress in the development of manually selected protoplasts of both accessions strongly depended on the volume of the protoplast-alginate suspension used for the feeder layer preparation. First symptoms of the development, i.e. an increase of cell size and the reorganization of cytoplasm, were observed in three-day-old cultures. The following cell divisions were observed in both volume variants of feeder layer systems, however, co-culture with a 200-µl feeder layer showed a better response. The first cell divisions were observed 2 days earlier (after 5 days of the culture) in comparison to a co-culture with a 100-µl feeder layer. Selected protoplasts cultured without a feeder layer (control variant), showed a slight increase in cell size and reorganization of the cytoplasm, although did not enter mitotic division. In 10-day-old cultures, clear and repeatable cell divisions in manually selected cells were observed only in co-culture with a 200-µl feeder layer, while in case of the co-culture with a 100-µl feeder layer, occasional cell divisions were noticed (Table 1). The further development of occasionally formed cell aggregates in a co-culture with a 100-µl feeder layer was stopped. A co-culture with a 200-µl feeder layer stimulated cell divisions, however, in 20-day-old cultures degradation of some cells was observed. In the following weeks multi-cell aggregates were formed in a co-culture with a 200-µl feeder layer. After 8 weeks of callus culture the development of somatic embryos was observed. No significant differences in the development of manually selected protoplasts derived from each donor accession were observed.

**Table 1:** The effect of feeder layer volume on development of manually selected leaf-derived protoplasts in two *Daucus* accessions

Source of manually selected protoplasts	Volume of feeder layer (µl)^a^	Mitotic divisions^b^ of manually selected protoplasts in			Somatic embryos^c^
		10-day-old cultures	20-day-old cultures		
cv. Dolanka	0	–	−		−
	100	±	−		−
	200	+	+ +		+
*D. carota* subsp. *gadecaei*	0	−	−		−
	100	±	−		−
	200	+	+ +		+

^a^Source of nurse protoplasts for feeder layer preparation was the same as for manual selection; feeder layer was formed from protoplast-alginate suspension with final protoplast density of 4 × 10^5^ per ml;

^b^(−) Cell divisions not observed; ( ±) occasional cell divisions, unrepeatable in subsequent repetitions; ( +) repeatable cell divisions of individual cells; (+ +) from manually selected protoplasts of proper morphology and undamaged membrane at least 50% re-entered mitotic divisions and cell aggregates were formed;

^c^(−) Not occurred; ( +) occurred; three independent experiments per each variant have been performed; in a single experiment 100 protoplasts per variant were manually selected and embedded in four alginate droplets (25 protoplasts per droplet)

### Development of the electrofusion procedure for leaf-derived protoplasts of carrot

The preliminary study on electrofusion parameters carried out in the micro-fusion chamber under direct microscopic control included two steps: (1) optimisation of alternating current (AC) for protoplast alignment and (2) optimisation of direct current (DC) for the reversible membrane breakdown and protoplast fusion.

**Fig. 3:**
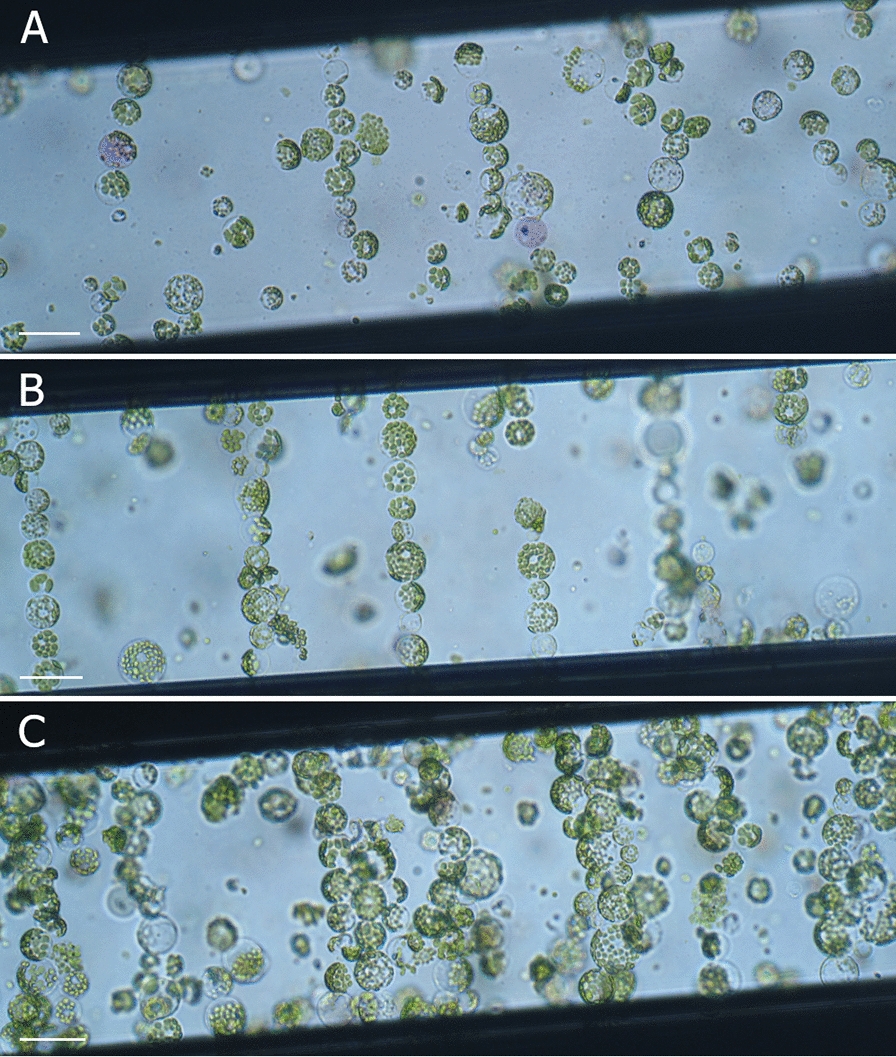
Protoplast chain formation during the alignment phase of electrofusion in the micro fusion chamber at (**A**) 125 V, 15 s; (**B**) 175 V, 15 s; (**C**) 200 V, 15 s; scale bar: 40 µm

The formation of the cell-chains was dependent both on applied AC voltage and duration of the pulse. The cell-chains were formed even if the lowest voltage of AC (100 or 125 V) was applied. However, in those conditions only 3–4 cells per chain were observed (Table 2; Fig. 3A) and larger cells were not aligned even during a 40 s pulse. Applying AC at voltages of 150 and 175 V for up to 10 s caused the formation of short cell chains, however unaligned single cells were also present. The application of 200 V AC caused the formation of grouped chains and protoplast deformation including changes in the cell shape and/or cell membrane disruption (Fig. 3C). The optimal parameters have been chosen, considering the level of protoplast deformation, as well as the length and protoplast arrangement in the chain. The longest single chains of undeformed cells were obtained when AC of 175 V was applied for 15 s (Fig. 3B).

**Table 2:** Effect of alternating current (AC) voltage and pulse duration on the protoplast-chain formation during the alignment phase of electrofusion between *D. carota* subsp. *sativus* cv. Dolanka and *D. carota* subsp. *gadecaei* protoplasts

AC voltage (V)	AC duration (s)	Cell no./chain, cell deformation^a^
100	10–40	3–4 cells, single chain, undeformed
125	10	3–4 cells, single chain, undeformed
	10–40	3–4 cells, single chain, undeformed
150	15–30	7–9 cells, single chain, undeformed
	40	4–7 cells, single chain, slightly deformed
175	10–20	8–11 cells, single chain, undeformed
	30–40	7–9 cells, single chain, slightly deformed
200	15	7–9 cells, grouped chains, slightly deformed
	15–40	7–9 cells, grouped chains, deformed

^a^Protoplast deformation manifested by changes in the cell shape or cell membrane disruption

For the efficient fusion frequency, the number of DC pulses, its voltage and duration should be estimated. The preliminary observations showed that a multiple fusion is promoted when more than one DC pulse is applied. Therefore, in the next experiments one DC pulse of different voltage (1.5–2.5 kV cm^−1^) and duration (40–100 µs) was tested (Table 3). After applying DC at a voltage of 1.5 kV cm^−1^, regardless of the pulse duration, fusion was incomplete and dumbbell shaped structures with incompletely fused membranes were observed. Such dumbbell shaped structures were also observed when DC at a voltage of 2.0–2.5 kV cm^−1^ and up to 70 µs was applied, while extending the pulse from 80 to 100 µs allowed complete fusion resulting in spherical-shaped cells. The level of cell damage was dependent on pulse duration—longer pulses increased the number of damaged cells. Additionally, a higher DC voltage combined with a longer pulse duration affected the increased frequency of multiple fusion. A high number of fusion events with relatively low protoplast damage was observed when DC at a voltage of 2 kV cm^−1^ from 70 to 90 µs was applied. Therefore, these parameters have been used during more detailed study.

**Table 3:** Effect of direct current (DC) voltage and pulse duration on the protoplast damage and cell fusion during the membrane breakdown phase of electrofusion between *D. carota* subsp*. sativus* cv. Dolanka and *D. carota* subsp. *gadecaei* protoplasts

DC voltage (kV cm^−1^)	Pulse duration (µs)	Protoplast damage^a^	Cell fusion^b^
1.5	40–100	−	±
2.0	< 70	−	±
	70	−	+
	80	+	+
	90	+	+
	100	+	+
2.5	< 70	−	±
	70	−	+
	80	+	+
	90	+ +	multiple
	100	+ +	multiple

^a^(-) Protoplast damage not observed; ( +) 1–5 damaged cells or (+ +) 6–10 damaged cells per microscopic field at 20 × magnification

^b^(±) Occasional cells showing incomplete fusion, unrepeatable in subsequent repetitions; (+) complete fusion occurred; multiple—fusions between 3 or more cells occurred

After applying DC at a voltage of 2.0 kV cm^−1^ from 70 to 90 µs, cells emitting green–red fluorescence were counted both in micro- and macro-fusion chambers to evaluate hetero-fusion frequency (Table 4). On average four times higher fusion frequency was observed in the micro-fusion chamber (with range 16–22%) in comparison to the macro-fusion chamber (with range 3–8%). In both cases, the highest frequency of hetero-fusion events (22 and 8%, respectively) was observed after applying DC for 90 µs.

**Table 4:** Effect of direct current (DC) pulse duration on hetero-fusion frequency between *D. carota* subsp. *sativus* cv. Dolanka and *D. carota* subsp. *gadecaei* protoplasts

Duration (µs) of 2 kV cm^−1^ DC	The frequency of hetero-fusion events (%)	
	Micro-fusion chamber	Macro-fusion chamber
70	19.3 ± 0.5 a	5.3 ± 0.6 b
80	15.9 ± 0.8 a	3.0 ± 0.1 c
90	21.6 ± 4.7 a	8.2 ± 0.5 a
Average	18.9 ± 1.6	5.5 ± 0.8

Data represent means and standard errors calculated from three independent fusion experiments per each fusion variant. Means within columns followed by the same letter were not significantly different at least at P = 0.05

Besides the hetero-fusion frequency, the effect of the electric field on (1) the protoplasts viability, (2) the ability to resynthesise the cell wall and (3) the ability to form cell aggregates (plating efficiency) was evaluated. The protoplast viability was dependent on duration of the pulse and ranged from 82% for control protoplasts (i.e. protoplast mix of both parental forms not treated with electric current) to 63% for protoplasts treated with DC at a voltage of 2.0 kV cm^−1^ for 90 µs (Table 5). Similarly, the ability to resynthesise the cell wall, evaluated in 72-h-old cultures, was influenced by the duration of DC pulses. The electrically treated protoplasts showed 14–20% lower frequency of cell wall resynthesis in comparison to the control. Differences in plating efficiency between the electrically treated protoplasts and the non-treated control were observed in 10-day-old cultures resulting in 11–23% lower number of forming cell aggregates. In 20-day-old cultures numbers of forming cell aggregates in electrically treated variants were lower in comparison to control but the differences were statistically not significant.

**Table 5:** Effect of direct current (DC) pulse duration on protoplasts viability, cell wall re-synthesis and plating efficiency in post-fusion mixture of *D. carota* subsp. *sativus* cv. Dolanka and *D. carota* subsp. *gadecaei* protoplasts

Duration (µs) of 2 kV cm^−1^ DC	Cell viability (%)^a^	Cell wall re-synthesis (%)^b^	Plating efficiency (%)^c^ in	
			10- day-old cultures	20-day-old cultures
Control	82.4 ± 1.3 a	90.0 ± 2.3 a	60.0 ± 3.1 a	63.1 ± 0.4 a
70	80.0 ± 3.1 a	76.5 ± 5.1 b	47.3 ± 3.7 ab	58.5 ± 4.2 a
80	76.1 ± 3.2 ab	69.6 ± 0.2 b	37.5 ± 6.4 ab	49.1 ± 5.2 a
90	63.1 ± 3.6 b	69.6 ± 1.4 b	49.4 ± 2.1 b	46.9 ± 2.8 a

^A^Evaluated just after protoplast embedding in alginate matrix;

^b^Frequency of the cells with completely reconstituted cell wall examined after calcofluor staining of cellulose in 72 h-old protoplast cultures;

^c^The number of cell colonies per total number of observed cells (× 100)

Data represent means and standard errors calculated from three independent fusion experiments per each fusion variant. Means within columns followed by the same letter were not significantly different at least at P = 0.05

Based on the collected data (Table 5), to produce hybrid cells in the macro-fusion chamber, cell alignment and reversible membrane breakdown were generated after applying AC at a voltage of 175 V for 15 s and DC at a voltage of 2.0 kV cm^−1^ for 90 µs, respectively.

### Selection of hybrid cells and regeneration of *D. carota* subsp. *sativus* ( +) *D. carota* subsp. *gadecaei* plants

**Fig. 4:**
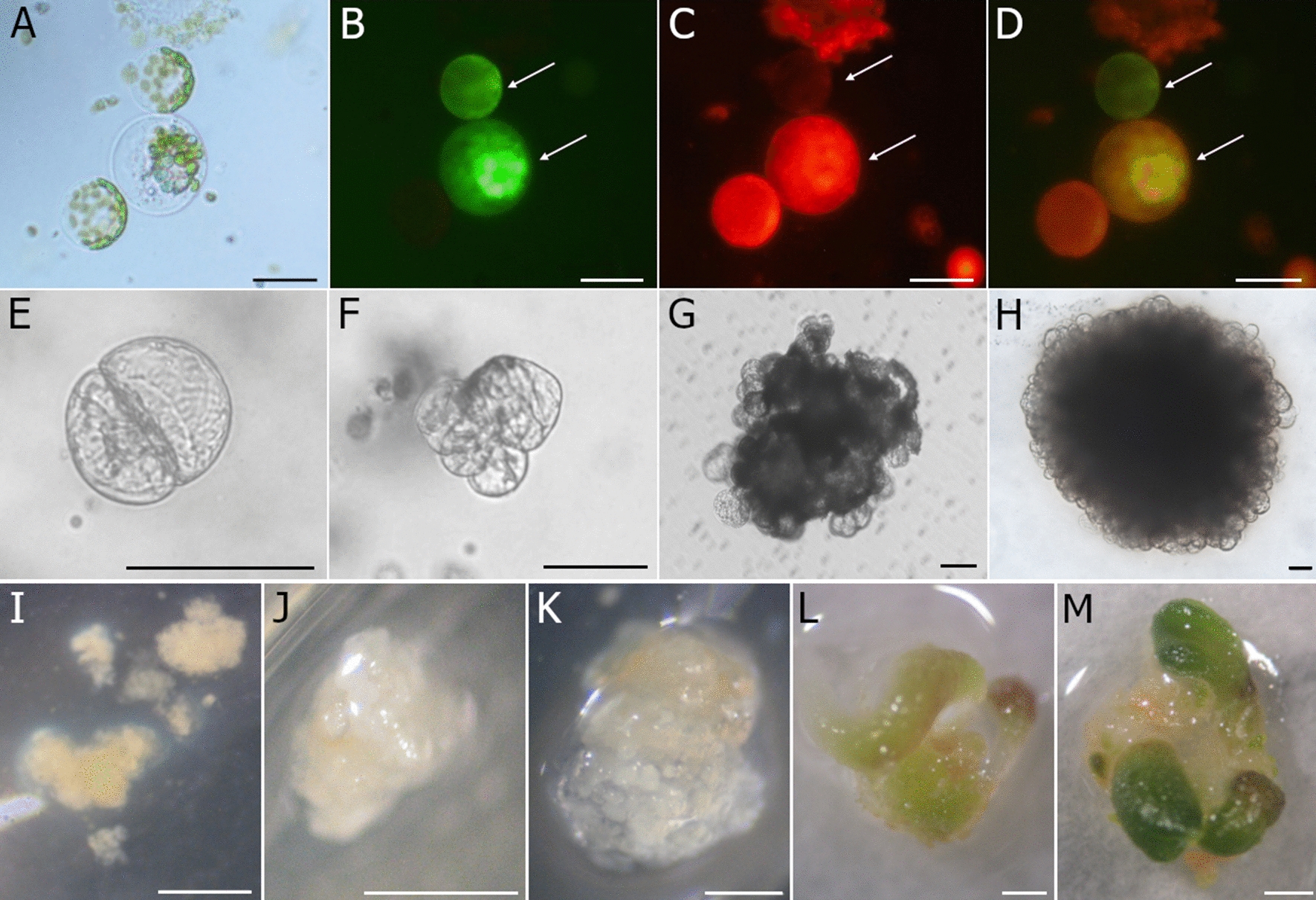
Protoplast-to-plant regeneration of ‘Dolanka’ ( +) *D. carota* subsp. *gadecaei* hybrids. (**A**–**D**) hybrid cells (**A**) emitting green (**B**) and red (**C**) fluorescence (marked by arrows); (**E**–**H**) development of a multicellular fusant-derived aggregate; (**I**–**M)** formation and development of somatic embryos observed in microcallus cultures derived from selected hybrid cells. Scale bars: 100 µm (**A**–**H**); 0.5 mm (I-L); 1 mm (**M**)

**Fig. 5:**
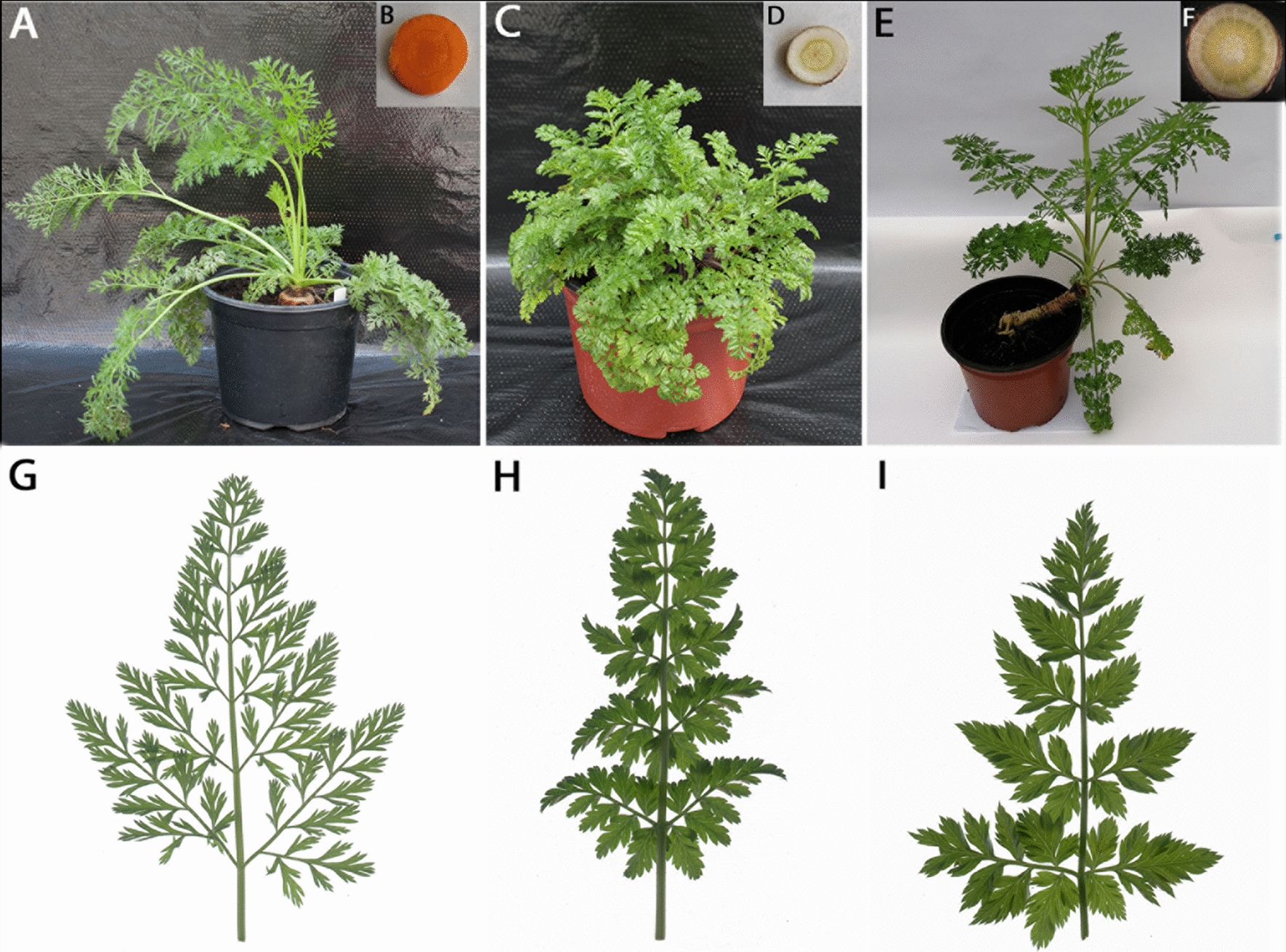
Phenotypic diversity observed for both parental components and obtained hybrids. (**A**–**F**) protoplast derived plants of *D. carota* subsp. *sativus* cv. Dolanka (**A**, **B**), *D. carota* subsp. *gadecaei* (**C**, **D**) and ‘Dolanka’ ( +) *D. carota* subsp. *gadecaei* hybrid (**E**, **F**); (**G**, **H**) leaf morphology observed for ‘Dolanka’ (**G**), *D. carota* subsp. *gadecaei* (**H**) and obtained hybrids (**I**)

Cells emitting green–red fluorescence were identified as hybrids (Fig. 4A–D), were manually selected, and transferred to alginate droplets. To stimulate their development, an established feeder layer system was applied successfully. In total, 429 putative hybrid cells have been selected and co-cultured with nurse protoplasts embedded in the feeder layer. In the first days of culture two types of morphological changes in putative hybrid cells were observed: (1) the increase in size and shape change from round to oval, or (2) the shrinking of cell membrane and cytoplasm. First cell divisions were observed from the 5th to the 10th day of culture (Fig. 4E), however, only about 50% of selected putative hybrid cells re-entered mitotic divisions. As a consequence of mitotic divisions, cell aggregates were formed (Fig. 4F–H). In some aggregates mitotic divisions were arrested over time and symptoms of cell death were observed. During two months of co-culture with a feeder layer, aggregates with mitotically active cells continued their growth leading to the formation of a microcallus (Fig. 4I). After approximately 2 weeks of the microcallus culture being on hormone-free medium, proembryonic mass and somatic embryos at different developmental stages (Fig. 4J–M) were observed. Somatic embryos usually regenerated into morphologically normal and rooted plants. Finally, 124 plants were regenerated and 92 of them were successfully acclimatised to *ex vitro* conditions. Regenerated putative hybrids showed phenotypic diversity as compared to parental forms (Fig. 5A–D). All somatic hybrids formed storage roots (Fig. 5E–F) similar to the cultivated carrot in size and shape, however, yellowish in colour as opposed to the orange root of *D. carota* subsp. *sativus* cv. Dolanka and white root of *D. carota* subsp. *gadecaei*. The leaves had an intermediate shape between the cultivated form and the wild component (Fig. 5G–I), however, all leaf blades were shiny as observed for *D. carota* subsp. *gadecaei.*

### Screening for molecular markers differentiating parental accessions and the identification of somatic hybrids

To facilitate the preliminary screening for markers differentiating parental accessions, DNA samples of both ‘Dolanka’ and *D. carota* subsp. *gadecaei* plants were pooled into four groups, each representing five genotypes of one accession. Created groups were genotyped using a panel of one hundred DcS-ILP markers. Amplification of nine markers resulted in the presence of unique DNA band patterns differentiating groups representing cv. Dolanka and *D. carota* subsp*. gadecaei*. Selected markers were then used to genotype each plant separately. Four markers (i.e. DcS-ILP214, DcS-ILP225, DcS-ILP516, and DcS-ILP906) differentiated both gene pools consistently. We used them for genotyping an additional twenty plants representing ‘Dolanka’ and *D. carota* subsp. *gadecaei* to confirm the repeatability of differentiation of cultivated carrot and wild *Daucus* subspecies, and to choose a marker(s) suitable for the identification of hybrid plants.

**Fig. 6:**
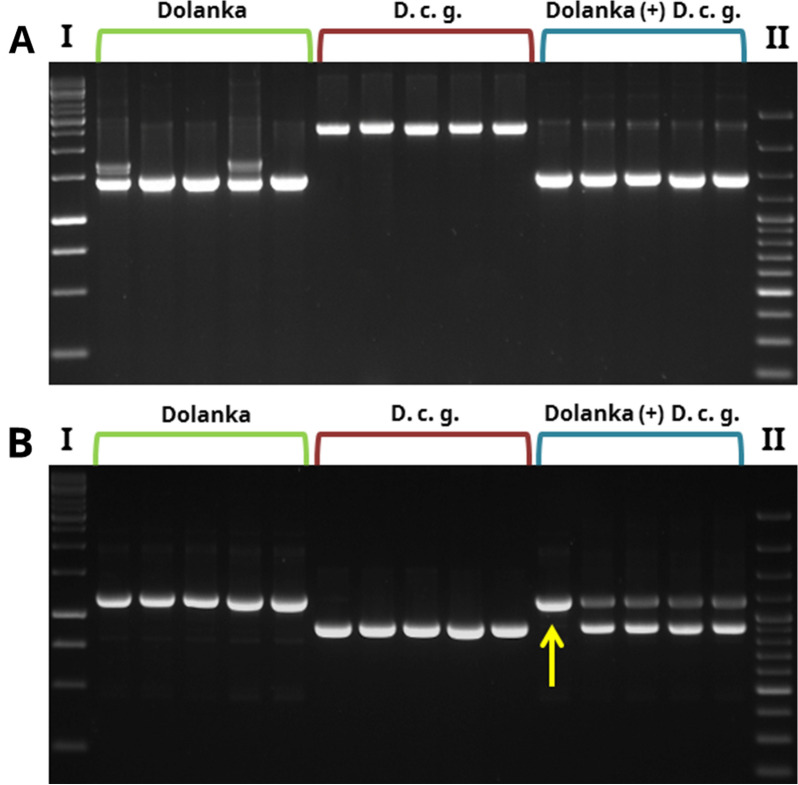
Amplification of DcS-ILP markers differentiating donors and identifying somatic hybrids of cv. Dolanka ( +) *Daucus carota* subsp. *gadecaei*. Data shown for five randomly chosen plants of cv. Dolanka (marked with a green bracket), *Daucus carota* subsp. *gadecaei* (*D.c.g*.; red bracket) and putative somatic hybrids (‘Dolanka’ ( +) *D.c.g.*; blue bracket). **A** Amplification of DcS-ILP225 characterized by the presence of ca. 1470 bp and 1730 bp specific products for ‘Dolanka’, ca. 2420 bp products for *D.c.g*., and both specific products for hybrid plants. **B** Amplification of DcS-ILP516 characterized by the presence of ca. 1180 bp specific product for ‘Dolanka’ and ca. 900 bp specific products for *D.c.g*. Hybrids were characterised by the presence of both specific products. One putative hybrid regenerated in vitro (Dx7-1) showed only one product specific for ‘Dolanka’ donor plant (marked by yellow arrow). I—GeneRuler 1 kb Plus DNA Ladder (Thermo Scientific^™^); II—GeneRuler 100 bp Plus DNA Ladder (Thermo Scientific^™^)

Amplification of DcS-ILP225 and DcS-ILP516 resulted in a clear distinction of cv. Dolanka and *D. carota* subsp. *gadecaei* gene pools. As predicted for codominant markers, DcS-ILP225 generated a maximum of two PCR products (ca. 1470 bp and 1730 bp) for ‘Dolanka’ genotypes and one PCR product (ca. 2420 bp) for *D. carota* subsp. *gadecaei* genotypes (Fig. 6A). Amplification of DcS-ILP516 resulted in the presence of ca. 1180 bp long PCR products for ‘Dolanka’ genotypes and ca. 900 bp long products for *D. carota* subsp. *gadecaei* genotypes (Fig. 6B). Non-specific PCR products were not observed on the electrophoregrams, thus, these two markers were chosen as the most suitable for the verification of putative ‘Dolanka’ ( +) *D. carota* subsp. *gadecaei* hybrids. The results of DcS-ILP214 and DcS-ILP906 amplification were not satisfying due to the presence of multiple products of variable size for ‘Dolanka’ plants (DcS-ILP214) and very small difference in the size (less than 30 bp) of PCR products obtained for ‘Dolanka’ and *D. carota* subsp. *gadecaei* (DcS-ILP906).

**Fig. 7:**
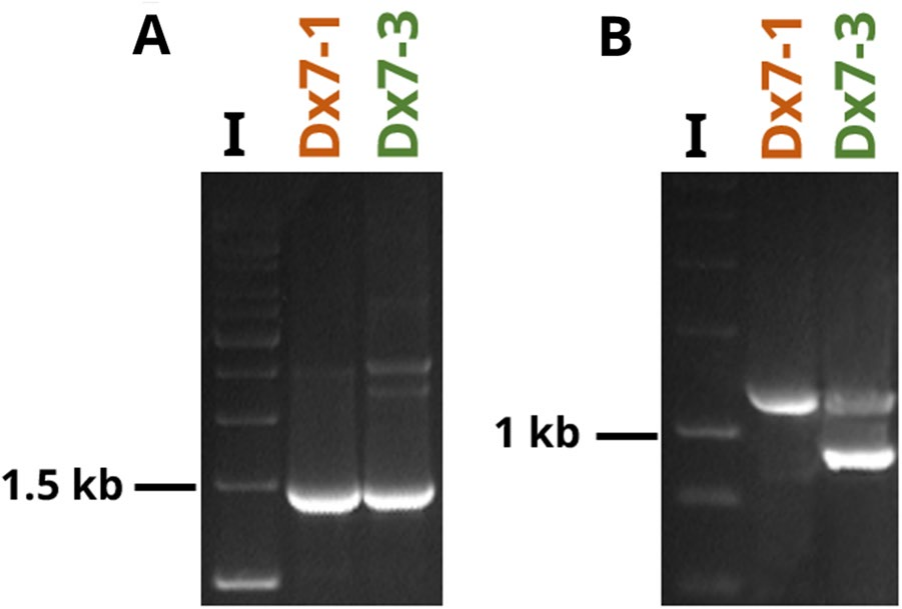
Amplification of DcS-ILP markers for two putative hybrids regenerated in vitro. **A** Amplification of DcS-ILP225 for plant Dx7-1 and Dx7-3. **B** Amplification of DcS-ILP516 for plant Dx7-1 and Dx7-3. In both loci (**A** and **B**) Dx7-1 plant carries alleles derived from only one donor (‘Dolanka’), whereas Dx7-3 shows the presence of alleles derived from both donors (‘Dolanka’ and *Daucus carota* subsp. *gadecaei*). I—GeneRuler 1 kb Plus DNA Ladder (Thermo Scientific^™^)

In total, 124 regenerated putative ‘Dolanka’ ( +) *Daucus carota* subsp. *gadecaei* hybrids were tested using DcS-ILP225 and DcS-ILP516 markers. One hundred twenty-three plants produced the expected PCR band patterns, suggesting the presence of genetic components derived from both donors. Amplification of DNA of one plant (namely Dx7-1) resulted in the presence of only one product, ca. 1470 bp long for DcS-ILP225 and ca. 1180 bp long for DcS-ILP516 (Fig. 7). This implicates that Dx7-1 is not a result of fusion of ‘Dolanka’ and *Daucus carota* subsp. *gadecaei* protoplasts.

## Discussion

**Fig. 8:**
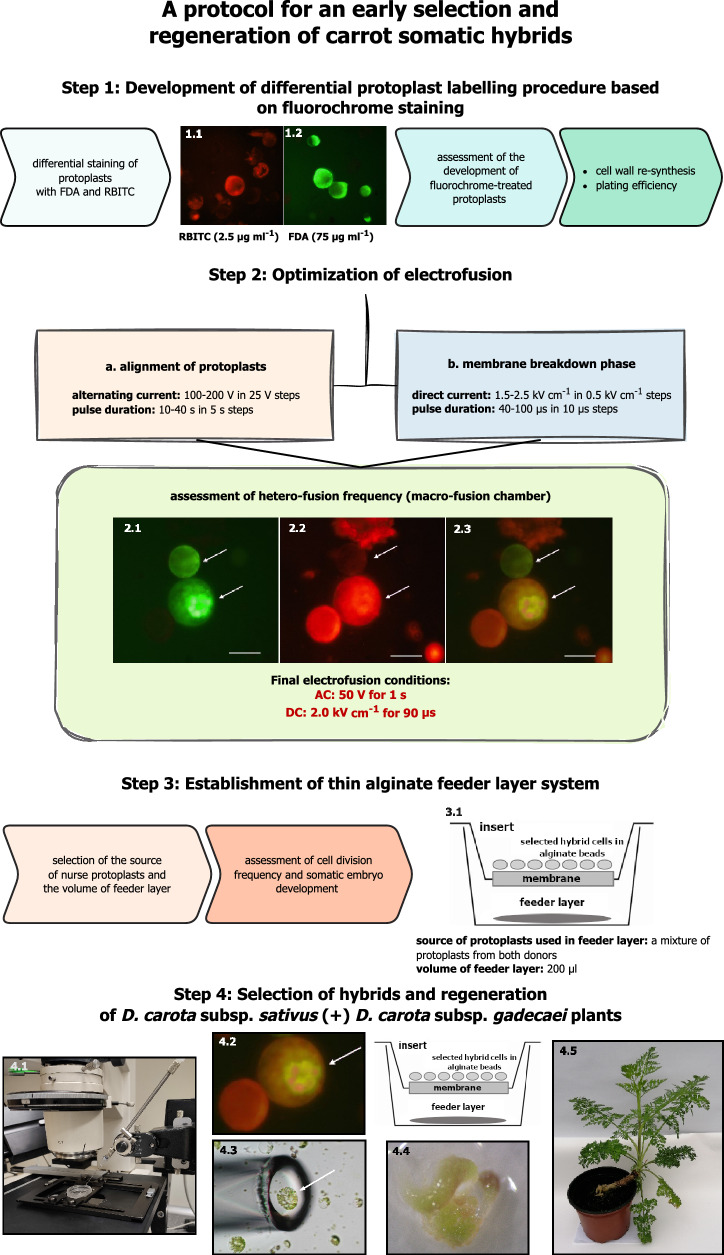
Flow chart showing four subsequent steps for the development of protocol for an early selection and regeneration of carrot somatic hybrids. (**1.1**) protoplast fluorescence after staining with rhodamine B isothiocyanate (RBITC); (**1.2**) protoplast fluorescence after staining with fluorescein diacetate (FDA); (**2.1**) fluorescence of FDA- and (**2.2**) RBITC- stained protoplasts as well (**2.3**) dual-colour fluorescence observed in the post-fusion protoplast mixture (putative hybrid cells are marked by arrows); (**3.1**) a schematic representation of the developed thin alginate feeder layer culture system; (**4.1**–**4.3**) a micromanipulator-based (4.1, 4.3) manual selection of dual-labelled hybrid cells (**4.2**); (**4.4**) development of somatic embryos observed in microcallus cultures derived from selected putative hybrid cells; (**4.5**) acclimatized to *ex vitro* conditions ‘Dolanka’ ( +) *D. carota* subsp. *gadecaei* hybrid. Scale bar: 100 μm

The present study was carried out to obtain the somatic hybrids in the genus *Daucus* and to develop efficient methods of their selection and regeneration (Fig. 8). Two subspecies, i.e. *Daucus carota* subsp. *sativus* and *Daucus carota* subsp. *gadecaei* have been chosen for several reasons. First, carrot serves as a model species for protoplast isolation and protoplast-to-plant regeneration. There are numerous reports of successful isolations of large numbers of highly viable protoplasts from leaf-derived tissue [14, 17, 18, 41]. Moreover, many carrot accessions are characterised by high regeneration capacity [14, 16, 20, 42–44]. The two selected subspecies not only have these features, but also display morphological differences, allowing for a preliminary identification of hybrids based on their phenotype. There are two requirements for a successful production of somatic hybrids: first, to establish an efficient method of protoplast fusion leading to a large number of viable hybrids, and second, to develop a procedure allowing for the selection of heterokaryons. Depending on the selection method, hybrids can be identified at different stages of development. Early selection is advantageous due to the reduction of space, resources and time required for the full regeneration of the entire pool of putative hybrids. It is also an essential step during production of somatic hybrids derived from the species characterised by very high regenerative ability, such as carrot, as the development of more frequent non-fusants might arrest growth of much less numerous heterokaryons in the post-fusion mixture. Hence, fluorescent or morphological markers can be applied directly after fusion. When mutants with the specific features are available, selection can be based on the different hormone requirements in a culture medium, the complementation of auxotrophic mutants or markers such as complementation between two chlorophyl-deficient mutants [3, 20, 45]. The main disadvantage of this method is the limited availability of mutation-bearing cell lines. Since there are no apparent differences in both size and morphology of *D. c.* subsp. *sativus* and *D. c.* subsp. *gadecaei* protoplasts and no mutation cell lines are available, the development of an efficient system for the identification of hybrids in the post-fusion mixture is essential.

### Development of dual fluorescence labelling of protoplasts for the effective detection of heterofusion products

In this study fluorescent labelling has been applied to overcome limitations of hybrid selections in a high-density post-fusion mixture. During the preliminary study, the intensity and efficiency of protoplast staining with three fluorescent labels i.e. fluorescein diacetate (FDA), rhodamine B isothiocyanate (RBITC) and scopoletin were evaluated. All three labels were previously shown as having no deleterious effect on the viability and growth of carrot cells [46–48]. We also did not observe a negative effect of the applied fluorochromes on the ability of protoplasts to resynthesise the cell wall. Fluorochrome-treated cultures efficiently regenerated into plants. Among the three labels, only two, FDA and RBITC, were chosen for the differential staining of protoplasts, as the identification of scopoletin-stained cells was very limited. Additionally, the scopoletin labelling required a whole night of incubation during enzymatic digestion, and a higher volume of the dye had been used in comparison to FDA and RBITC. Despite this, there were no differences in the efficiency of protoplast isolation between scopoletin and FDA or RBITC stained tissues.

### Establishment of the thin alginate feeder layer system for the development of manually selected protoplasts

The proposed protocol for the fluorescence-based selection of hybrids leads to the isolation of a relatively small number of heterokaryons from the post-fusion mixture. Therefore, a low-density protoplast culture technique needs to be applied for the proper development of hybrids. The culture density is a known factor affecting development of protoplasts and the optimal effective density for most of studied plant species is between 5 and 100 × 10^4^ cells/ml [49]. To support the growth of target cells in a low-density culture, a feeder layer can be established [50, 51]. The use of feeder cells to induce cell divisions of protoplasts obtained from species considered as recalcitrant, or cells cultured in low density was previously reported for cauliflower, banana and sugar beet [52–54]. In this study, the feeder layer protocol has been developed to effectively overcome the limitations of culturing a small number of putative hybrids selected from the post-fusion mixture. The mixture of protoplasts obtained from both donors, i.e. cv. Dolanka and subspecies *gadecaei*, proved to be the most suitable as the nurse culture. The most significant effect on the growth promotion of target cells was observed for the ratio of target cells to feeder cells of approximately 1:800. Lower density of feeder cells resulted in a limited frequency of mitotic divisions and ultimately led to cell growth arrest in the 20th day of the culture, most probably due to the insufficient concentration of growth promoting factors. To increase the rate of cell colony formation and to avoid cell agglutination, both hybrid cells and feeder cells were embedded in thin layers of alginate. This method of cell immobilization has been successfully used for many species including thale cress, cabbage, canola, love-in-a-Mist and carrot [17, 55–59]. The beneficial effect of physical separation and immobilization on cells might result from a better oxygen supply, lower ethylene levels and a stable change of osmotic pressure in the first step of the protoplast culture [2, 60–62].

### Development of the electrofusion procedure for leaf-derived protoplasts of carrot

Protoplast fusion can be induced via various treatments. Chemical fusion and electrofusion are among the most effective methods for protoplast fusion [26]. Both methods have been successfully implemented in *Daucus* family [8, 42, 47, 63] but electrofusion, mostly due to an easier control of fusion parameters, is becoming more prevalent. The high yield of electrofusion depends on many biological, chemical, and physical factors including pulse parameters, tissue source for protoplasts, cell size and medium used for fusion. In this study, we focused on the analysis of physical parameters of electrofusion, as it is essential to establish a balance between fusion frequency and viability of protoplasts. An efficient electrofusion can be achieved only when cell membranes are in close contact, and this contact is maintained until a pulse can be applied [29]. The changes in membrane potential and protoplast close contact can be obtained by the application of an alternating electric field (AC). The contact between fused cells needs to be strong and the contact area should be as large as possible [64]. The optimal results in terms of a sufficient chain cell formation and reduction of cell damage were obtained by applying an AC field of 175 V for 15 s. These parameters are in correspondence with other studies carried out on carrot protoplasts. Nea and Bates [65] routinely applied AC in the range of 160–200 V in their studies assessing factors affecting protoplast electrofusion efficiency, whereas Gieniec et al. [63] applied 175 V in their protocol for a real-time detection of somatic hybrid cells of carrot. Moreover, we proved that the developed parameters can be applied for both donors as no differences in cell response were observed for cv. Dolanka and *D. carota* subsp. *gadecaei*. The protoplasts showed no signs of deformation and formed single chains, which is crucial for frequent fusion events. The formation of binucleate heterokaryons is favoured when single chain cells are formed [66]. Application of direct current pulses (DC) on adhered protoplast cause reversible membrane breakdown resulting in pores in membranes and then membrane fusion [64, 67]. DC of 2 kV cm^−1^ applied for 90 µs proved to be sufficient for a reversible membrane breakdown of mesophyll-derived protoplasts and complete membrane fusion leading to the formation of heterokaryons. Although the use of lower voltage but longer direct current pulses might result in lowered cell viability after fusion, the fusion efficiency was satisfactory. In their work, Gieniec et al. [63] performed electrofusion of carrot protoplasts characterised by similar viability as in the present study (78% vs. 82%). The parameters of electrofusion differed with regard to applied DC (2.5 to 3 kV cm^−1^ for 50 µs in [63] vs. 2 kV cm^−1^ for 90 µs in this study). The cell viability assessed after the fusion substantially dropped when longer pulses were applied (71% in [63] vs. 63% in the present study), but both the fusion efficiency (0.9–13.9% in [63] vs. 16–22% in the present study) and the plating efficiency (16.6% in [63] vs. 49.4% in the present study) were substantially higher when lower voltage was applied.

### Selection of hybrid cells and regeneration of *D. carota* subsp. *sativus* ( +) *D. carota* subsp. *gadecaei* plants

Observation of dual-colour fluorescence in the post-fusion protoplast mixture indicated the presence of two fused components. The fusion frequencies obtained in the present experiment permitted a manual isolation of moderate numbers of heterokaryons. Approximately 50% of isolated hybrid cells re-entered mitotic divisions and formed aggregates. This relatively high percentage of fusants with arrested cell cycle is not uncommon, as the plating efficiency reported by e.g. [68] and [63] were much lower (0.01–0.1% and 16.6%, respectively). This could be possibly attributed to the irreversible cell damage that occurred during the electrofusion or cell transfer or might be an effect of chromosomal translocations and/or substitutions that followed the fusion event. As often observed for carrot [17, 18, 43], obtained microcallus cultured on hormone-free medium developed into proembryonic mass and further into somatic embryos. Seventy four percent of regenerants were acclimatised to *ex vitro* conditions which is comparable to the results presented for cv Dolanka by Kiełkowska et al. [18]. In the greenhouse conditions, somatic hybrid plants were as vigorous as the parental lines. Similarly to the work of Dudits et al. [43], morphological traits intermediate between cv. Dolanka and *D. carota* subsp*. gadecaei* provided evidence strongly supporting the fact that the ninety-two regenerants were somatic hybrids.

### Screening for molecular markers differentiating parental accessions and the identification of somatic hybrids

But even when the hybrid cells were subjected to selective pressure or physically isolated, the hybrid nature of the regenerated plants should be confirmed, as escapes from the selective conditions and/or misclassification of hybrid cells cannot be excluded a priori. In the present study we report a successful use of the intron length polymorphism (ILP) analysis in order to validate the hybrid status of 124 regenerants obtained from selected hybrid cells. Using two codominant ILP markers located on carrot chromosome 2 and 5 we were able to repeatably distinguish both parent components and identify somatic hybrids among regenerants. This method of validation showed a very high correlation with the dual-label fluorescence approach—99% of regenerated plants originating from dual-fluorescent cells were confirmed hybrids.

## Conclusions

In the presented study, we demonstrated a method for the transfer of the genome of wild subspecies of carrot to cultivated carrot via electrofusion. We used a dual-labelling fluorescence approach for the early selection of hybrid cells and confirmed its accuracy by the means of molecular analysis of the regenerated plants. Moreover, the early selection of hybrids, performed just after fusion and achieved by dual labelling of fusants combined with manual selection of heterokaryons, proved to be a suitable approach for species with a high regenerative ability, such as carrot, as it allows for regeneration of almost exclusively hybrid cells. The obtained hybrids might serve as a valuable material for the study of carrot genetics and breeding. The developed methods of fusion, selection of hybrid cells and detection of hybrid status can be used to obtain somatic hybrids with valuable agronomic traits within *Daucus* genus. Wild carrot has a great potential to widen the genetic diversity through somatic hybridization. Therefore, this technique may serve as an alternative to combine the genomes and to transfer nuclear or cytoplasmatic traits from wild *Daucus* species to cultivated carrot.

## Methods

### Plant material

As a protoplast source, two accessions of *Daucus* (carrot) have been used i.e. cultivated form of *Daucus carota* L. subsp. *sativus* Hoffm. cv. Dolanka and wild subspecies of carrot i.e. *Daucus carota* L. subsp. *gadecaei* (Rouy & E. G. Camus) Heywood (Table 6). Protoplasts were isolated from young plants germinated from seeds in i*n vitro* conditions. For this purpose, seeds of donor accessions were surface disinfected according to the three-step procedure described by Grzebelus et al. [14], including incubation in: 40 ℃ water bath, 0.2% (*v*/*v*) solution of fungicide ‘Bravo’ (Syngenta, Waterford, Ireland), 20% (*w*/*v*) water solution of chloramin T (sodium *N*-chlorotoluene-4-sulphonamide), 30 min each, and three rinses with sterile distilled water. Then seeds were placed on Murashige and Skoog (MS) medium [69] (Duchefa Biochemie, The Netherlands), supplemented with 30 *g *l^−1^ sucrose, solidified with 6.5 *g *l^−1^ plant agar (Biocorp, Poland) and maintained at 18 ± 2 ℃ in the dark for germination. After 7 days, seedlings were transferred to glass jars with R medium composed of MS macro- and micro-elements, 0.1 mg l^−1^ thiamine HCl, 0.1 mg l^−1^ pyridoxine HCl, 0.5 mg l^−1^ nicotinic acid, 3.0 mg l^−1^ glycine, 100 mg l^−1^ myo-inositol, 20 *g *l^−1^ sucrose, and 2.5 *g *l^−1^ phytagel (Sigma, USA) and kept in a climate room at 26 ± 2 ℃ under 16-h photoperiod and light intensity of 55 μmol m^−2 ^s^−1^ (fluorescent lamps Sylvania Gro-lux T8, USA).

**Table 6:** Seed source and somatic chromosome number (2n) of *Daucus* accessions used for protoplast cultures

Accession	Seed source	2n
*D. carota* subsp. *sativus*	Poland, OP cv. Dolanka	18
*D. carota* subsp. *gadecaei*	UK, HRI 7160	18

Genetic Resources Unit (Wellesbourne, UK) number, 2n somatic chromosome number

*OP* open-pollinated, *HRI* Horticulture Research International,

### Protoplast isolation

Protoplasts were isolated from leaves with petioles of 2‒4-week-old in vitro grown plants following the procedure described by Grzebelus et al. [14]. Briefly, 1 *g* of plant material was cut into pieces, pre-treated in 8 ml of the plasmolysis solution (0.5 M mannitol; Sigma) and then incubated in 8 ml of the enzyme solution composed of 1% (w/v) cellulase Onozuka R-10 (Duchefa), 0.1% (w/v) pectolyase Y-23 (Duchefa), 20 mM MES [2-(N-Morpholino)ethanesulfonic acid] (Sigma), 5 mM CaCl_2_ (Sigma), and 0.6 M mannitol for 16 h on a gyratory shaker (30 rpm; Rotamax 120, Heidolph Instruments, Germany) at 26 ± 2 ℃ in the dark. Then the protoplasts were separated from undigested tissues by filtration through a nylon mesh (80–100 µm; Millipore, USA) and centrifuged (100 *g* for 5 min; MPW-223e, MPR Med Instruments, Poland; rotor type: MPR no 12,485). The pellet was resuspended in 8 ml of solution containing 0.5 M sucrose and 1 mM MES and overlaid with 2 ml of the W5 solution [70] for gradient centrifugation (145 *g* for 10 min). Viable protoplasts localized in the interphase between sucrose and W5 solution were transferred into a fresh tube, washed two times by centrifugation in W5 solution (100 *g* for 5 min each) and left on ice until further processing.

### Development of dual fluorescence labelling of protoplasts

To select appropriate set of fluorochromes for labelling of parental protoplasts before fusion procedure, fluorochromes with different spectral properties were tested *i.e.* emitting green fluorescence (fluorescein diacetate, FDA; Sigma), red fluorescence (rhodamine B isothiocyanate, RBITC; Sigma) and blue fluorescence (scopoletin; Sigma). Due to the limited availability of *D. carota* subsp. *gadecaei* seeds, the optimization of protoplast labelling was carried out on protoplasts isolated only from cultivated carrot ‘Dolanka’. To find out the proper conditions for stable and strong fluorescence of labelled protoplasts two concentrations of FDA (15 and 75 µg ml^−1^) and RBITC (1.25 and 2.5 µg ml^−1^) were examined. For that purpose, working solutions of FDA and RBITC were prepared by dissolving 30 µl or 150 µl of 0.5% FDA acetone stock solution (Table 7) and 2.5 µl or 5 µl of 0.5% RBITC stock solution in 10 ml 0.4 M mannitol. Then the individual working solutions were mixed with purified protoplasts and incubated in the dark, at room temperature for 10 min. In case of scopoletin five concentrations were examined *i.e.*: 25, 50, 75, 100 and 125 µg ml^−1^. Scopoletin staining was carried out during an overnight enzymatic digestion of the donor tissue by applying appropriate volume of 0.1% scopoletin stock solution (Table 7) to enzyme solution.

**Table 7:** Stock solutions of fluorochromes used for protoplast labelling and cellulose detection

Fluorochrome	Solvent	Concentration (%)
Fluorescein diacetate (FDA)	Acetone	0.5
Rhodamine B isothiocyanate (RBITC)	dH_2_O	0.5
Scopoletin	0.6 M mannitol (pH = 9.0)	0.1
Calcofluor white (CW)	NaOH + *d*H_2_O (pH = 10–11)	1.0

All stock solutions were filter-sterilized (0.22 µm, Millipore) and stored in aliquots in – 20 ℃

In all labelling variants, after incubation with individual fluorochromes, protoplast suspension was purified from unbound dye molecules by centrifugation in cold 0.4 M mannitol (pH 5.8) from 1 to 4 times (100 *g* for 5 min each). After washing step, protoplasts were carefully resuspended in mannitol solution, mixed in ratio 1:1 (v/v) in three pairs of colour variants (*i.e.* (1) scopoletin- with RBITC-stained protoplasts, (2) scopoletin- with FDA-stained protoplasts and (3) FDA- with RBITC –stained protoplasts) and incubated on ice for 1 h. The protoplast mixture was then observed under a fluorescent microscope (Axiovert S100, Carl Zeiss, Germany) to assess whether unbound dye particles were completely removed during the washing step, and thus, no cells emitting dual fluorescence were visible before fusion treatment. The final stage of the protoplast labelling procedure involved the selection of the most suitable pair of fluorochromes for further detection of hybrid cells. During this step the following characteristics were taken into account: intensity of fluorescence, presence of the background signal, retention of dye in the cells and lack of dye diffusion from solution to protoplasts.

### Effect of fluorochromes on protoplast development

Both, stained and control (unstained) ‘Dolanka’ protoplasts were suspended in 0.4 M mannitol and then their density was adjusted to 8 × 10^5^ protoplasts per ml. Prepared protoplast suspensions were mixed in ratio 1:1 (v/v) with 2.8% filter-sterilized alginate solution (Sigma; [17]) and 300 µl protoplast-alginate mixture was gelated (as thin alginate layer) in 6 cm Petri dishes on Ca-agar medium composed of 1% (w/v) plant agar (Biocorp), 20 mM CaCl_2_ and 0.4 M mannitol for 1 h at room temperature. In the next step, alginate layers were transferred into 6 cm Petri dishes containing 4 ml CPP medium (according to [14]) additionally supplemented with 200 nM PSK (phytosulfokine-α, PeptaNova GmbH, Germany) and 200 mg l^−1^ cefotaxime (Polfa Tarchomin SA, Poland), hereinafter referred to as protoplast culture medium (PCM). Cultures were incubated at 26 ± 2 ℃ in the dark for about two months.

In order to evaluate the effect of fluorochromes on protoplast development, the ability to re-synthesis the cell wall and the plating efficiency were evaluated. To identify newly synthesized cell wall, staining of cellulose with calcofluor white (CW; Sigma) was applied to 48-h-old protoplast cultures. Briefly, 10 µl of 1% filter-sterilized CW water stock solution (Table 7) was added to 4 ml of PCM in Petri dish with protoplasts embedded in alginate matrix. After 10 min of incubation medium was replaced for the fresh one. The observations of cellulose deposition were performed under an inverted microscope equipped with the filter set appropriate for detection of blue fluorescence of calcofluor-stained cellulose. Frequency of cells with newly synthesized cell-wall was determined and expressed as a percentage of cells with completely or partially reconstructed cell wall out of total observed cells. The plating efficiency, defined as the ability of single cells to form colonies through continuous mitotic divisions, was assessed on the 10th and 20th day of culture and presented as the number of cell colonies per the total number of observed undivided cells and cell aggregates.

### Establishment of thin alginate feeder layer system for development of manually selected protoplasts

Protoplasts of both parental components were used as nurse protoplasts in preparation of the feeder layer. For this purpose, after the purifying step, protoplast density was adjusted to 8 × 10^5^ cells per ml. Then protoplasts of both parental forms were mixed in ratio 1:1 (v/v) and added to an equal volume of 2.8% filter-sterilized alginate solution [17]. For the feeder layer preparation, 100 or 200 µl aliquots of nurse protoplast-alginate mixture were gelated as thin alginate layers in 6 cm Petri dishes on Ca-agar medium (see section *Effect of fluorochromes on protoplast development*) for 30 min at room temperature and then placed in a well of the 6-well multi-dish plate containing 4 ml of PCM, as described above. Protoplasts of cv. Dolanka or *D. carota* subsp. *gadecaei* were picked up from appropriate protoplast suspension using manual TransferMan NK2 micromanipulator (Eppendorf, Germany) coupled with an inverted microscope. For the selection, an oil micro-injector equipped with 75 µm needles (Stripper Tips, Origio, Denmark) was used. About 25 selected protoplasts were transferred to 20 µl droplet of alginate-PCM mixture (1:1, v/v) placed on Ca-agar medium. Four solidified droplets with embedded protoplasts were placed into a sterile insert with transparent PET (polyethene terephthalate) membrane of 8 µm pore size and density 6 ± 2 × 10^4^ cm^−2^ (BD Bioscience, USA), immersed into a well of the 6-well multi-dish plate containing PCM, and co-cultured with nurse protoplasts embedded in a thin alginate layer. Cultures were incubated at 26 ± 2 ℃ in the dark for about two months.

### Development of the electrofusion procedure for leaf-derived protoplasts of carrot

The preliminary experiments for establishment of electrofusion parameters were carried out in the micro-fusion chamber (Eppendorf, electrode distance of 0.2 mm) connected to the proper insert of the multiporator (Eppendorf). An aliquot of 20 µl of protoplast suspension in 0.4 M mannitol was pipetted on both electrodes of the fusion chamber placed under an inverted microscope and the fusion events were monitored directly after applying an electric field. The alignment phase was optimised for the mixture of parental protoplasts, while the phase of membrane breakdown was monitored separately for each parental component. The voltage pulse setup and its duration for the alignment phase was performed by the use of alternating current (AC) within a range from 100 to 200 V in 25 V steps. Additionally, the time of cell-chain formation was tested in a range from 10 to 40 s with 5 s steps. During these experiments, the number of cells per chain and level of cell deformation manifested by changes in the cell shape or cell membrane disruption were determined. The phase of membrane breakdown was set up by the use of direct current (DC) within a range from 1.5 to 2.5 kV cm^−1^ in 0.5 kV cm^−1^ steps and pulse duration with a range from 40 to 100 µs in 10 µs steps. During this set of experiments, both the occurrence of complete fusion and the level of protoplast damage were monitored. After the phase of membrane breakdown, the post-alignment phase has been applied by the use of AC at a voltage of 50 V for 1 s. To track cell behaviour in an electric field and to evaluate the frequency of hetero-fusion, FDA- and RBITC-stained protoplasts were used, as described in section: *Development of dual fluorescence labelling of protoplasts*. Protoplasts of both parental components at the density of 4 × 10^5^ cells per ml, stained separately with each fluorescent dye, were mixed in a ratio of 1:1 and then transferred to the micro-fusion chamber. After applying the electric field, the fused (i.e. emitting dual-colour fluorescence) and non-fused (i.e. emitting one-colour fluorescence) protoplasts were visualised using proper filter sets mounted to the microscope. Images from subsequent fluorescent channels were acquired and the frequency of dual-colour (green–red) cells was assessed based on the merged images from both channels using ImageJ software [71]. Microscopic observations of the cell behaviour in the applied electric field served as a starting point for the optimization of electrofusion parameters in macro scale (i.e. in the macro-fusion chamber). For that purpose, FDA- and RBITC-stained protoplasts were mixed in equal volumes and 250 µl of the protoplast suspension was carefully pipetted to the bottom of the Helix macro-fusion chamber (Eppendorf, electrode distance of 0.2 mm), then slowly screwed with the insert electrode to avoid formation of air bubbles, and connected to the proper insert of the multiporator. Electrofusion parameters were the same as applied in the micro-fusion chamber. After electric field treatment protoplasts were incubated in the fusion chamber on ice for 20 min. Then the suspension of fused and non-fused protoplasts (post-fusion mixture) was carefully transferred to 0.5 ml Eppendorf tube and diluted with cold 0.4 M mannitol in ratio 1:3 (v/v). After that, 100 µl of post-fusion mixture was transferred to a 3.5 cm Petri dish and placed under the inverted microscope. The frequency of green–red hybrids was counted and expressed as a percentage of green–red protoplasts out of total observed cells.

In order to evaluate the effect of the electric field on cell development, non-stained protoplasts at the density of 10^6^ for each parental component were fused in a macro-fusion chamber, mixed in a ratio of 1:1 with sodium alginate solution (2.8%) and placed on Ca-agar medium. After gelation, protoplasts embedded in alginate layers were cultured in 6 cm Petri dishes with 4 ml of PCM. To assess the effect of the electric field on protoplasts (1) the cell’s viability, (2) the cell’s ability to resynthesise the cell wall, and (3) the plating efficiency were evaluated. The viability of cells was evaluated just after embedding in alginate matrix, by staining with FDA as described by Grzebelus et al. [14], and expressed as a percentage of cells with green fluorescence out of the total observed cells. To identify newly synthesised cell wall, staining of cellulose with CW was applied to 72-h-old protoplast cultures (for staining details see section *Effect of fluorochromes on protoplast development*) and frequency of cells with completely reconstructed cell wall was determined. Plating efficiency was assessed on the 10th and 20th day of culture.

### Manual selection of hybrid cells

After electrofusion, the post-fusion mixture was transferred to the 0.5 ml tube and kept on ice until the selection of hybrids. Before the manual selection, the post-fusion mixture was diluted in a ratio of 1:3 with 0.4 M cold mannitol to avoid agglutination of protoplasts. An aliquot of 100 µl has been transferred to the 3.5 cm Petri dish with a polymer coated bottom (µ-Dish, Ibidi, Germany). The covered Petri dish was placed under an inverted microscope (in unsterile conditions) and the lid was removed immediately before inserting the sterile micromanipulator needle. Hybrid cells systematically emitting dual green–red fluorescence were identified using fluorescent mode of the microscope and picked up by the needle. The 20 µl droplet of alginate-PCM mixture (1:1) was placed on the Ca-agar medium in 6 cm Petri dish. The covered Petri dish was transferred onto the microscopic table, placed close to the needle holder of micromanipulator and the collected hybrids were gently immersed into the alginate droplet in such a way that the exposure time to unsterile conditions was as short as possible. The procedure was repeated until the cells in the post-fusion mixture aggregated. About 25 selected cells were inserted into one alginate droplet.

### Hybrids culture, regeneration, and acclimatisation

After 20 min gelation of alginate, manually selected protoplasts were cultured as described in the section *Establishment of the thin alginate feeder layer system for the development of manually selected protoplasts*. After 2 months of co-culture with the feeder layer prepared from the suspension of ‘Dolanka’ and *D. carota* subsp. *gadecaei* protoplasts, alginate droplets with tissue developed from selected putative hybrid cells were placed on filter paper, washed in 20 mM sodium citrate solution with 0.2 M mannitol (pH = 5.8; [14]), then in CPPD medium containing 0.1 mg l^−1^ α-naphthaleneacetic acid (NAA), 0.2 mg l^−1^ zeatin, pH = 5.6 [13] and placed on solidified R medium in 9 × 2.5 cm Petri dish (START™DISH, de Ville Biotechnology, Poland). The cultures covered with double filter paper were maintained in a climate room at 26 ± 2 ℃ under 16-h photoperiod and light intensity of 55 μmol m^−2^ s^−1^. Two weeks later, the filter paper was removed, regenerating tissues were transferred onto fresh R medium and kept in the same conditions as mentioned above. Fully developed plants were transplanted to the moss-coconut fibre substrate (Ceres International, Poland) and acclimatised to *ex vitro* conditions for 2 weeks in a climate chamber (MLR-352H, SANYO, Japan) at 18 ± 1 ℃ under 16-h photoperiod and light intensity of 30 μmol m^−2^ s^−1^. In the 1st week, the relative humidity was adjusted to 90% while in the second-week humidity was reduced by 2% every day. During the acclimatisation plants were watered moderately.

### Identification of somatic hybrids using the DcS-ILP molecular marker system

To verify putative hybrids a PCR-based molecular marker system was chosen. We exploited transposition-based panel of DcS-ILP markers (*Daucus carota Stowaway* Intron Length Polymorphism markers) developed by Stelmach et al. [38]. Total genomic DNA of the tested plant was isolated from fresh young leaves using a modified CTAB protocol [72]. The identification of somatic hybrids was a two-stage process. In the first step, DNA of twenty ‘Dolanka’ plants, nineteen *D. carota* subsp. *gadecaei* plants, and a plant representing doubled haploid line (DH1—the reference genotype; see [38]) were used for the screening for DcS-ILP markers systematically differentiating the parental accessions. Then, in the second step, one hundred twenty-four putative ‘Dolanka’ ( +) *D. carota* subsp. *gadecaei* plants were genotyped using selected differentiating DcS-ILP markers to identify somatic hybrids. PCRs, electrophoresis and recording of electrophoretic bands were carried out as described by Stelmach et al. [38].

### Microscopic observations and data analysis

All microscopic analyses were performed under an inverted Axiovert S100 microscope (Carl Zeiss, Germany) using bright-field illumination or fluorescence mode conjugated with the appropriate Zeiss filter sets for FDA (filter set 16: λex = 485/20 nm, λem = 515 nm), RBITC (filter set 14: λex = 510–560 nm, λem = 590 nm) and scopoletin/calcofluor (filter set 02: λex = 365 nm, λem > 420 nm). Microscopic data acquisition was proceeded with a PowerShot G10 camera (Canon, Japan) and processed with AxioVision 4.8 (Carl Zeiss MicroImaging) and ImageJ software [71].

All data were collected in three independent experiments with a single treatment represented by three Petri dishes per repetition. Counts were carried out on 100–200 cells per single Petri dish. The mean values and standard errors were calculated. The overall effect of treatments was assessed using analysis of variance (ANOVA) with Tukey’s honestly significant difference (HSD) test to determine differences between the means. Significant differences were expressed at least at P = 0.05. The computations were performed using Statistica ver. 12 (StatSoft. Inc. 2014).

## Data Availability

The datasets supporting the conclusions of this article are included within the article or are available from corresponding author on reasonable request.

## References

[1] Schenk RU, Hildebrandt AC (1968). Somatic hybridization: a new approach to genetic change. Am J Bot.

[2] Pati PK, Sharma M, Ahuja PS (2008). Rose protoplast isolation and culture and heterokaryon selection by immobilization in extra thin alginate film. Protoplasma.

[3] Krumbiegel G, Schieder O (1979). Selection of somatic hybrids after fusion of protoplasts from *Datura innoxia Mill*. and *Atropa belladonna L.*. Planta.

[4] Horita M, Morohashi H, Komai F (2003). Production of fertile somatic hybrid plants between oriental hybrid lily and *Lilium* x *formolongi*. Planta.

[5] Tiwari JK, Devi S, Ali N, Luthra SK, Kumar V, Bhardwaj V (2017). Progress in somatic hybridization research in potato during the past 40 years. Plant Cell Tiss Org.

[6] Begum F, Paul S, Bag N, Sikdar SR, Sen SK (1995). Somatic hybrids between *Brassica juncea* (L). Czern and *Diplotaxis harra* (Forsk.) Boiss and the generation of backcross progenies. Theor Appl Genet.

[7] Matsumoto K, Duarte Vilarinhos A, Oka S (2002). Somatic hybridization by electrofusion of banana protoplasts. Euphytica.

[8] Kisaka H, Kisaka M, Kanno A, Kameya T (1997). Production and analysis of plants that are somatic hybrids of barley (*Hordeum vulgare* L.) and carrot (*Daucus carota* L.). Theor Appl Genet.

[9] Johnson AT, Veilleux RE, Jules J (2001). Somatic hybridization and applications in plant breeding. Plant breeding reviews.

[10] de Bona CM, de Carvalho DC, Stelly DM, Creighton Miller J, Louzada ES (2011). Symmetric and asymmetric somatic hybridization in citrus: review. Citrus Res Technol.

[11] Grambow HJ, Kao KN, Miller RA, Gamborg OL (1972). Cell division and plant development from protoplasts of carrot cell suspension cultures. Planta.

[12] Kameya T, Uchimiya H (1972). Embryoids derived from isolated protoplasts of carrot. Planta.

[13] Dirks R, Sidorov V, Tulmans C (1996). A new protoplast culture system in *Daucus carota* L. and its applications for mutant selection and transformation. Theor Appl Genet.

[14] Grzebelus E, Szklarczyk M, Baranski R (2012). An improved protocol for plant regeneration from leaf- and hypocotyl-derived protoplasts of carrot. Plant Cell Tiss Org.

[15] Sivanesan I, Hayward A, Bandaralage JH, O’brien CM, Ranaware AS, Kunchge NS (2023). Protoplast technology and somatic hybridisation in the family Apiaceae. Plants.

[16] Grzebelus E, Skop L (2014). Effect of β-lactam antibiotics on plant regeneration in carrot protoplast cultures. In Vitro Cell Dev Biol.

[17] Maćkowska K, Jarosz A, Grzebelus E (2014). Plant regeneration from leaf-derived protoplasts within the *Daucus* genus: effect of different conditions in alginate embedding and phytosulfokine application. Plant Cell Tiss Org.

[18] Kiełkowska A, Grzebelus E, Lis-Krzyścin A, Maćkowska K (2019). Application of the salt stress to the protoplast cultures of the carrot (*Daucus carota* L.) and evaluation of the response of regenerants to soil salinity. Plant Cell Tiss Org.

[19] Bruznican S, Eeckhaut T, Van Huylenbroeck J, De Keyser E, De Clercq H, Geelen D (2021). An asymmetric protoplast fusion and screening method for generating celeriac cybrids. Sci Rep.

[20] Kisaka H, Lee H, Kisaka M, Kanno A, Kang K, Kameya T (1994). Production and analysis of asymmetric hybrid plants between monocotyledon (*Oryza sativa* L.) and dicotyledon (*Daucus carota* L.). Theor Appl Genet.

[21] Tanno-Suenaga L, Ichikawa H, Imamura J (1988). Transfer of the CMS trait in *Daucus carota* L. by donor-recipient protoplast fusion. Theor Appl Genet.

[22] Han L, Zhou C, Shi J, Zhi D, Xia G (2009). Ginsenoside Rb1 in asymmetric somatic hybrid calli of *Daucus carota* with *Panax quinquefolius*. Plant Cell Rep.

[23] Cocking EC (1960). A method for the isolation of plant protoplasts and vacuoles. Nature.

[24] Takebe I, Labib G, Melchers G (1971). Regeneration of whole plants from isolated mesophyll protoplasts of tobacco. Naturwissenschaften.

[25] Wallin A, Glimelius K, Eriksson T (1974). The induction of aggregation and fusion of *Daucus carota* protoplasts by polyethylene glycol. Z Pflanzenphysiol.

[26] Kao KN, Michayluk MR (1974). A method for high-frequency intergeneric fusion of plant protoplasts. Planta.

[27] Schaeffer P, Cami B, Hotchkiss RD (1976). Fusion of bacterial protoplasts. Proc Natl Acad Sci USA.

[28] Pontecorvo G (1975). Production of mammalian somatic cell hybrids by means of polyethylene glycol treatment. Somatic Cell Genet.

[29] Zimmermann U, Scheurich P (1981). High frequency fusion of plant protoplasts by electric fields. Planta.

[30] Bates GW, Hasenkampf CA (1985). Culture of plant somatic hybrids following electrical fusion. Theor Appl Genet.

[31] Blackhall NW, Davey MR, Power JB, Dixon RA, Gonzales RA (1994). Applications of protoplast technology. Plant cell culture: a practical approach.

[32] Barsby TL, Yarrow SA, Shepard JF (1984). Heterokaryon identification through simultaneous fluorescence of tetramethylrhodamine isothiocyanate and fluorescein isothiocyanate labelled protoplasts. Stain Technol.

[33] Durieu P, Ochatt SJ (2000). Efficient intergeneric fusion of pea (*Pisum sativum* L.) and grass pea (*Lathyrus sativus* L.) protoplasts. J Exp Bot.

[34] Geerts P, Druart P, Ochatt SJ, Baudoin JP (2008). Protoplast fusion technology for somatic hybridisation in *Phaseolus*. Biotechnol Agron Soc Environ.

[35] Nair AS, Chee HT, Schwarzacher T, Harrison PH (2005). Genome classification of banana cultivars from South India using IRAP markers. Euphytica.

[36] Li S, Ramakrishnan M, Vinod KK, Kalendar R, Yrjälä K, Zhou M (2019). Development and deployment of high-throughput retrotransposon-based markers reveal genetic diversity and population structure of asian bamboo. Forests.

[37] Singh S, Nandha PS, Singh J (2017). Transposon-based genetic diversity assessment in wild and cultivated barley. Crop J.

[38] Stelmach K, Macko-Podgórni A, Machaj G, Grzebelus D (2017). Miniature inverted repeat transposable element insertions provide a source of intron length polymorphism markers in the carrot (*Daucus carota* L.). Front Plant Sci.

[39] Branco CJS, Vieira EA, Malone G, Kopp MM, Malone E, Bernardes A (2007). IRAP and REMAP assessments of genetic similarity in rice. J Appl Genet.

[40] Queen RA, Gribbon BM, James C, Jack P, Flavell AJ (2004). Retrotransposon-based molecular markers for linkage and genetic diversity analysis in wheat. Mol Genet Genom.

[41] Godel-Jędrychowska K, Maćkowska K, Kurczyńska E, Grzebelus E (2019). Composition of the reconstituted cell wall in protoplast-derived cells of *Daucus* is affected by phytosulfokine (PSK). Int J Mol Sci.

[42] Dudits D, Maroy E, Praznovszky T, Olah Z, Gyorgyey J, Cella R (1987). Transfer of resistance traits from carrot into tobacco by asymmetric somatic hybridization: Regeneration of fertile plants. Proc Natl Acad Sci USA.

[43] Dudits D, Hadlaczky G, Lévi E, Fejér O, Haydu Z, Lázár G (1977). Somatic hybridisation of *Daucus carota* and *D. capillifolius* by protoplast fusion. Theor Appl Genet.

[44] Ichikawa H, Tanno-Suenaga L, Imamura J (1987). Selection of *Daucus cybrids* based on metabolic complementation between X-irradiated *D*.* capillifolius* and iodoacetamide-treated *D*.* carota* by somatic cell fusion. Theor Appl Genet.

[45] Kisaka H, Kameya T (1994). Production of somatic hybrids between *Daucus carota* L. and *Nicotiana tabacum*. Theor Appl Genet.

[46] Kanchanapoom K, Brightman AO, Grimes HD, Boss WF (1985). A novel method for monitoring protoplast fusion. Protoplasma.

[47] Kanchanapoom K, Boss WF (1986). The effect of fluorescent labeling on calcium-induced fusion of fusogenic carrot protoplasts. Plant Cell Rep.

[48] Widholm JM (1972). The use of fluorescein diacetate and phenosafranine for determining viability of cultured plant cells. Stain Technol.

[49] Eeckhaut T, Lakshmanan PS, Deryckere D, Van Bockstaele E, Van Huylenbroeck J (2013). Progress in plant protoplast research. Planta.

[50] Smith JA, Green CE, Gengenbach BG (1984). Feeder layer support of low density populations of *Zea mays* L. suspension cells. Plant Sci Lett.

[51] Ludwig SR, Somers DA, Petersen WL, Pohlman RF, Zarowitz MA, Gengenbach BG (1985). High frequency callus formation from maize protoplasts. Theor Appl Genet.

[52] Walters TW, Earle ED (1990). A simple, versatile feeder layer system for *Brassica oleracea* protoplast culture. Plant Cell Rep.

[53] Hall RD, Pedersen C, Krens FA (1993). Improvement of protoplast culture protocols for *Beta vulgaris* L. (sugar beet). Plant Cell Rep.

[54] Assani A, Chabane D, Foroughi-Wehr B, Wenzel G (2006). An improved protocol for microcallus production and whole plant regeneration from recalcitrant banana protoplasts (*Musa* spp.). Plant Cell Tiss Org.

[55] Klimek-Chodacka M, Kadluczka D, Lukasiewicz A, Malec-Pala A, Baranski R, Grzebelus E (2020). Effective callus induction and plant regeneration in callus and protoplast cultures of *Nigella damascena* L.. Plant Cell Tiss Org.

[56] Kiełkowska A, Adamus A (2021). Exogenously applied polyamines reduce reactive oxygen species, enhancing cell division and the shoot regeneration from *Brassica oleracea* L. var. *capitata* protoplasts. Agronomy.

[57] Kiełkowska A, Adamus A (2019). Peptide growth factor phytosulfokine-α stimulates cell divisions and enhances regeneration from *B oleracea* var. *capitata* L. protoplast culture. J Plant Growth Regul.

[58] Sahab S, Hayden MJ, Mason J, Spangenberg G (2019). Mesophyll protoplasts and PEG-mediated transfections: transient assays and generation of stable transgenic canola plants. Methods Mol Biol.

[59] Jeong YY, Lee HY, Kim SW, Noh YS, Seo PJ (2021). Optimization of protoplast regeneration in the model plant *Arabidopsis thaliana*. Plant Methods.

[60] Lian YJ, Lin GZ, Zhao XM, Lim HT (2011). Production and genetic characterization of somatic hybrids between leaf mustard (*Brassica juncea*) and broccoli (*Brassica oleracea*). In Vitro Cell Dev Biol.

[61] Bajaj YP, Bajaj YP (1989). Recent advances in the isolation and culture of protoplasts and their implications in crop Improvement. Plant protoplasts and genetic engineering I.

[62] Kanwar K, Bhardwaj A, Deepika R (2009). Efficient regeneration of plantlets from callus and mesophyll derived protoplasts of *Robinia pseudoacacia* L. Plant Cell Tiss Org.

[63] Gieniec M, Siwek J, Oleszkiewicz T, Maćkowska K, Klimek-Chodacka M, Grzebelus E (2020). Real-time detection of somatic hybrid cells during electrofusion of carrot protoplasts with stably labelled mitochondria. Sci Rep.

[64] Zimmermann U (1983). Electrofusion of cells: principles and industrial potential. Trends Biotechnol.

[65] Nea L, Bates G (1987). Factors affecting protoplast electrofusion efficiency. Plant Cell Rep.

[66] Bates G, Chang D, Chassy B, Saunders J, Sowers A (1992). Electrofusion of plant protoplasts and the production of somatic hybrids. Guide to electroporation and electrofusion.

[67] Cullis PR, Hope MJ (1978). Effects of fusogenic agent on membrane structure of erythrocyte ghosts and the mechanism of membrane fusion. Nature.

[68] Yamamoto T, Nakajima Y, Oeda K (2000). Morphological changes in homeotic cytoplasmic male-sterile carrots combined with fertile cytoplasm by asymmetrical cell fusion. Plant Cell Rep.

[69] Murashige T, Skoog F (1962). A revised medium for rapid growth and bio assays with tobacco tissue cultures. Physiol Plant.

[70] Menczel L, Nagy F, Kiss Z, Maliga P (1981). Streptomycin resistant and sensitive somatic hybrids of *Nicotiana tabacum* + *Nicotiana knightiana*: correlation of resistance to *N. tabacum* plastids. Theor Appl Genet.

[71] Schneider C, Rasband W, Eliceiri K (2012). NIH Image to ImageJ: 25 years of image analysis. Nat Methods.

[72] Briard M, Clerc V, Grzebelus D, Senalik D, Simon PW (2000). Modified protocols for rapid carrot genomic DNA extraction and AFLP™ analysis using silver stain or radioisotopes. Plant Mol Biol Report.

